# The Enhanced Catalytic Activities of Asymmetric Au-Ni Nanoparticle Decorated Halloysite-Based Nanocomposite for the Degradation of Organic Dyes

**DOI:** 10.1186/s11671-016-1252-9

**Published:** 2016-02-06

**Authors:** Lei Jia, Tao Zhou, Jun Xu, Xiaohui Li, Kun Dong, Jiancui Huang, Zhouqing Xu

**Affiliations:** Department of Physics and Chemistry, Henan Polytechnic University, Jiaozuo, 454000 People’s Republic of China; State Key Laboratory of Solid Lubrication, Lanzhou Institute of Chemical Physics, Chinese Academy of Sciences, Lanzhou, 730000 People’s Republic of China

**Keywords:** Janus Au-Ni nanoparticle, Halloysite nanotube, Dye degradation, Magnetic nanocomposite

## Abstract

Janus particles (JPs) are unique among the nano-/microobjects because they provide asymmetry and can thus impart drastically different chemical or physical properties. In this work, we have fabricated the magnetic halloysite nanotube (HNT)-based HNTs@Fe_3_O_4_ nanocomposite (NCs) and then anchored the Janus Au-Ni or isotropic Au nanoparticles (NPs) to the surface of external wall of sulfydryl modified magnetic nanotubes. The characterization by physical methods authenticates the successful fabrication of two different magnetic HNTs@Fe_3_O_4_@Au and HNTs@Fe_3_O_4_@Au-Ni NCs. The catalytic activity and recyclability of the two NCs have been evaluated considering the degradation of Congo red (CR) and 4-nitrophenol (4-NP) using sodium borohydride as a model reaction. The results reveal that the symmetric Au NPs participated NCs display low activity in the degradation of the above organic dyes. However, a detailed kinetic study demonstrates that the employ of bimetallic Janus Au-Ni NPs in the NCs indicates enhanced catalytic activity, owing to the structurally specific nature. Furthermore, the magnetic functional NCs reported here can be used as recyclable catalyst which can be recovered simply by magnet.

## Background

During recent years, the concepts of Janus particles are relatively new in nanoscience [1, 2]. Much work have been made to fabricate asymmetric particles due to their potential applications in a variety of fields such as catalysis [3], optical imaging [4], or biological applications [5, 6]. Moreover, the investigations of organic–inorganic nanocomposite materials also attracted people’s enthusiasm for research during the past decades [7, 8]. In general, tubular systems usually exhibit superior aerodynamic and hydrodynamic properties than the nanospheres [9]. Otherwise, materials combining inorganic nanotubes and well-defined polymers can be utilized as catalysts [10, 11], nanocontainers [12], carriers of drug or enzyme [13, 14], scavenging agents [15], and others [16].

The tubular clay minerals occupy a special place and they are readily available at low cost. The environmental friendly and biocompatible halloysite nanotubes (HNTs) have been acknowledged as rising star in materials science due to lots of advantages [17, 18]. Recently, HNTs used as a substrate for the organization of noble metal nanoparticles excitingly attract interest for many potential applications which due to their unique optical, electronic, imaging, magnetic, and catalytic properties [19, 20].

**Fig. 1:**
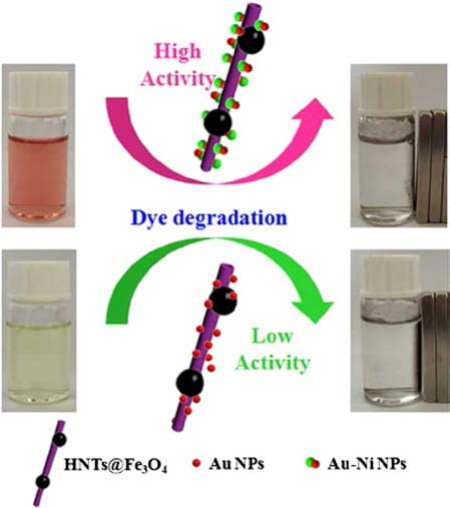
Schematic illustration of the catalytic reduction of organic dyes by using HNTs@Fe_3_O_4_@Au and HNTs@Fe_3_O_4_@Au-Ni nanocomposites as the catalysts

However, the studies on the catalytic activities of the Janus NPs loaded natural nanoclays are relatively less. The adsorption process of the Janus NPs to the area of the nanoclays often makes the nature of the Janus catalysts changed from hydrophobic to hydrophilic and therefore can be used in the catalytic degradation of some toxic dyes such as Congo red and 4-nitrophenol in water. Here, we report the simple fabrications of two multicomponent nanocatalysts HNTs@Fe_3_O_4_@Au and HNTs@Fe_3_O_4_@Au-Ni, which are composed by the hydrophilic magnetic halloysite clay nanotubes and the symmetric or Janus nanoparticles. Briefly, the magnetic HNTs@Fe_3_O_4_ nanotubes are fabricated through hydrothermal method by simply mixing the inorganic metal salt and halloysite clay nanotubes. Then, the symmetric Au or asymmetric Au-Ni NPs are deposited on the external wall of sulfydryl modified HNTs@Fe_3_O_4_ via the metal-S bonds. The catalytic activity and cycling stability of HNTs@Fe_3_O_4_@Au and HNTs@Fe_3_O_4_@Au-Ni NCs are investigated chosen the reduction of Congo red and 4-nitrophenol with NaBH_4_ as a model reaction. The results suggest that the as-prepared HNTs@Fe_3_O_4_@Au nanocomposite has low catalytic activity while the HNTs@Fe_3_O_4_@Au-Ni nanocomposite reveals excellent catalytic property and cycling stability, which can be potentially applied as recyclable and low-cost catalytic materials (as shown in Fig. 1).

## Experimental Methods

### Materials

3-Mercaptopropyl trimethoxysilane (MPTMS), octadecylamine (ODA), oleic acid (OA), vanillin, HAuCl_4_⋅4H_2_O, sodium acetate, FeCl_3_, and Ni(NO_3_)_2_⋅6H_2_O were obtain from the Chemical Reagent Co. of Shanghai (Shanghai, China). Congo red (CR) and 4-nitrophenol were purchased from Alfa Aesar and used without further purification. The kaolinite used in this study was provided by China-Kaolinite Company (China). Janus Au-Ni NPs and asymmetric Au NPs were synthesized according to literature [21, 22]. Chloroform and other solvents were analytical grade from Beijing Chemical Factory (China) and were used without further purification. Ultrapure water used in all experiments was obtained from a NANO Pure Infinity System (Barnstead/Thermolyne Corp.).

### Synthesis of Superparamagnetic HNTs@Fe_3_O_4_ Nanocomposite

In a typical procedure, the synthesis of Fe_3_O_4_ nanoparticles was carried out by modified reduction reactions between FeCl_3_ and ethylene glycol in the solvothermal system described in the literature [13, 23]. The experimental details about the synthesis of the nanocomposite were as follows: 1 g FeCl_3_⋅6H_2_O was dissolved in 30 mL of ethylene glycol to form a clear solution. Then, 2.7 g of sodium acetate and 0.75 g of polyethylene glycol were added with constant stirring for 30 min. After that, nanoclays (0.3 g) were ultrasonically dispersed in the resulting dispersion for 3 h. The mixture was sealed in a Teflon-lined stainless steel autoclave (50 mL capacity) and maintained at 200 °C for 8 h. Then, the mixture was cooled to ambient temperature. The obtained black magnetite particles were washed with ethanol and deionized water in sequence and dried in vacuum at 60 °C for 24 h.

### Surface Modification of Thiol-Terminated HNTs@Fe_3_O_4_

Two hundred microliters of MPTMS was added to 30 mL of toluene solution containing 100 mg of as-synthesized HNTs@Fe_3_O_4_ NPs, and the mixture was refluxed for 10 h. The resulting NPs were collected by centrifugation and washed several times with ethanol and dried overnight in a vacuum at 50 °C.

### Fabrication of HNTs@Fe_3_O_4_@Au or HNTs@Fe_3_O_4_@Au-Ni Nanocomposite

In a typical procedure, the as-functionalized HNTs@Fe_3_O_4_ NPs were suspended in 15 mL of chloroform at a concentration of 1 mg/mL and purged with argon for 5 min. Then, 15 mL of degassed chloroform containing Au or Au-Ni NPs was added (1 mg/mL), and the solution was reacted for 6 h in a shaker. After reaction completion, the resulting samples were collected using a magnet without washing and investigated by transmission electron microscopy (TEM).

### Evaluation of Catalytic Activity and Recovery Capability of Congo Red by the Nanocomposited Catalysts

Ten milligrams of the HNTs@Fe_3_O_4_@Au or HNTs@Fe_3_O_4_@Au-Ni was added into 100 mL of the CR solution (20 mg/L) with 0.0568 g of NaBH_4_. After set time intervals, the nanocomposites were instantly separated from the solution by suction filtering through a filter, and the UV–vis spectra of the solution were scanned at 25 °C in a range of 200–800 nm and the absorbance was determined. The change of absorbance was used as a criterion to evaluate the reduction efficiency. The used nanocomposites were recycled by using a magnet without washing and then reused to catalytic reduction of CR dye as the similar procedure described above. The recovery process was repeated for 10 cycles, and the change of decoloration efficiency for CR solution within 10 min was used to indicate the recovery capability of the nanocomposites.

### Evaluation of Catalytic Activity and Recovery Capability of 4-Nitrophenol by the Nanocomposited Catalysts

The catalytic reduction of 4-nitrophenol with NaBH_4_ was accomplished as follows: a 0.10 mmol/L 4-nitrophenol aqueous solution (1.50 mL) and 10.0 mmol/L NaBH_4_ aqueous solution (1.50 mL) were put in a quartz cell for UV–vis spectroscopy, 10 mg HNTs@Fe_3_O_4_@Au or HNTs@Fe_3_O_4_@Au-Ni nanocomposite was then added to the quartz cell, and the UV–vis absorption spectra were recorded immediately after mixing.

### Characterization

TEM was carried out using a JEOL 2100FX at acceleration voltages of 200 kV. One drop of suspension was drop-casted onto a carbon-coated copper TEM grid. Upon solvent evaporation, the sample was used for TEM observation without further treatment. X-ray powder diffraction (XRD) patterns were collected on a X′pert PRO X-ray power diffractometer (PAN analytical Co., Netherlands) using Cu Ka radiation of 1.5406 A (40 kV, 30 mA). The surface elemental analysis was conducted using an Energy Dispersive Analysis System of X-ray (EDX) (GENESIS, EDAX). The UV spectra were recorded on a Shimadzu UV-240 spectrophotometer.

## Results and Discussion

**Fig. 2:**
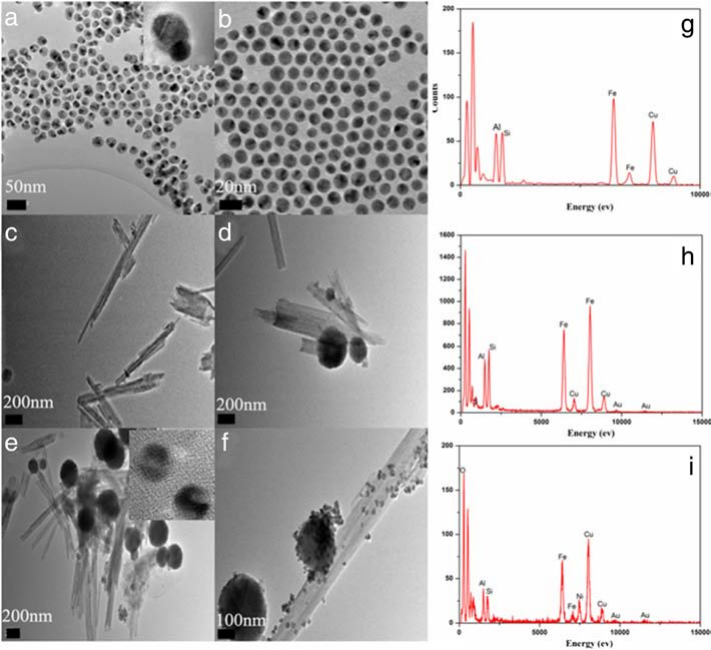
TEM images of Au-Ni (**a**), Au (**b**), pure HNTs (**c**), HNTs@Fe_3_O_4_ (**d**), HNTs@Fe_3_O_4_@Au (**e**), HNTs@Fe_3_O_4_@Au-Ni (**f**), and the EDX spectrum of pure HNTs (**g**), HNTs@Fe_3_O_4_@Au (**h**) and HNTs@Fe_3_O_4_@Au-Ni (**i**); *inset* pictures of **a** and **e** are the HRTEM images of the Au-Ni and Au NPs

The TEM image (Fig. 2a) displays the spindle-like morphology of the Janus Au-Ni hybrid nanocrystals. From the top right corner of Fig. 2a, we can see the tip size of the Au domain is ∼10 nm, and the tail diameter of the Ni domain is ∼20 nm. Meanwhile, the symmetric Au nanoparticles have a diameter of 10 nm, which also show good dispersibility (Fig. 2b). As shown in Fig. 2c, TEM image of original HNTs reveals that HNTs is cylindrical-shaped tube with multilayer walls and an open-ended lumen along the nanotube [18].

As described above, the HNTs have a two-layer structure, which are held together via hydrogen bonds, dipolar interactions, and attractive van der Waals forces [24]. As the outside of the kaolinites show negative charges, the iron(III) cations in the solution will likely to attach to the surface of kaolinites and then were reduced to nanoparticles under hydrothermal conditions [25, 26]. As shown in Fig. 2d, the Fe_3_O_4_ has been successfully located to the surfaces of the single kaolinite tube or between two kaolinite tubes. While simply blending the Au or Au-Ni NPs with thiol group modified HNTs@ Fe_3_O_4_ NPs, the symmetric Au or asymmetric Au-Ni particles will arrange on the surface of HNTs@Fe_3_O_4_, generating the HNTs@Fe_3_O_4_@Au (Fig. 2e) and HNTs@Fe_3_O_4_@Au-Ni (Fig. 2f) nanocomposites. Compositional analysis by energy dispersive X-ray analysis (EDX) indicates the presence of Fe after the recombination of pure clays and FeCl_3_, and the Au, Ni after the surface modification and adsorption processes (Fig. 2g–i). The smaller intensity of the Au peaks in EDX spectra is associated with smaller concentration of the metallic gold presenting in the sample.

**Fig. 3:**
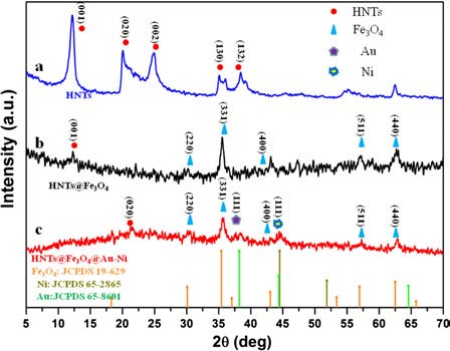
XRD pattern of pure HNTs, HNTs@Fe_3_O_4_, and HNTs@Fe_3_O_4_@Au-Ni nanocomposites

Figure 3a shows the XRD patterns of pure clays, some characteristic diffraction peaks can be assigned to the (001), (020), (002), (130), and (132) planes [13], while the peaks at 45°–65° range are from clay impurities (quartz, alunite). Figure 3b shows the XRD patterns of different morphologies of HNTs@Fe_3_O_4_ NPs. Five resolved peaks at 2θ = 30.08°, 35.51°, 43.08°, 57.01°, and 62.56° can be assigned as the fcc Fe_3_O_4_. Therefore, it also further confirms that HNTs@Fe_3_O_4_ nanocomposite has been successfully synthesized. Figure 3c shows the XRD pattern of as-obtained HNTs@Fe_3_O_4_@Au-Ni nanocomposite, which clearly indicates the cubic-phase Au (JCPDS 65-8601) and cubic-phase Ni (JCPDS 65-2865) and the nature of heterodimer structures of Au-Ni NPs.

**Fig. 4:**
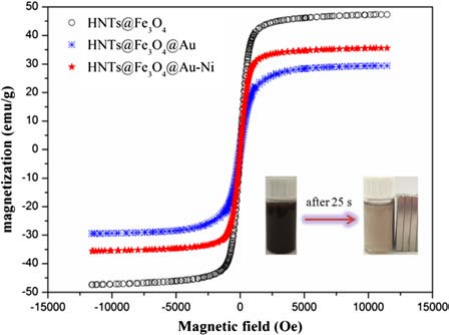
The magnetic hysteresis loops of HNTs@Fe_3_O_4_, HNTs@Fe_3_O_4_@Au, and HNTs@Fe_3_O_4_@Au-Ni nanocomposites

The magnetic hysteresis loops of the samples shown in Fig. 4 reveal that the samples are ferromagnetic at room temperature. The saturation magnetization of HNTs@Fe_3_O_4_ nanocomposite is 47.31 emu/g. However, owing to the existence of Au NPs loaded on the surfaces of clays, the saturation magnetization of HNTs@Fe_3_O_4_@Au nanocomposite decreases to 29.43 emu/g. While after loading the magnetic Janus Au-Ni NPs, the saturation magnetization of HNTs@Fe_3_O_4_@Au-Ni decreases to 35.82 emu/g, which is similar to the reported examples [27]. As shown in the inset of Fig. 4, the magnetic HNTs@Fe_3_O_4_@Au-Ni nanocomposite shows fast response (25 s) to the external magnetic field because of their high magnetization, which suggests this nanocatalyst can be easily separated by magnet after the catalytic reactions.

**Fig. 5:**
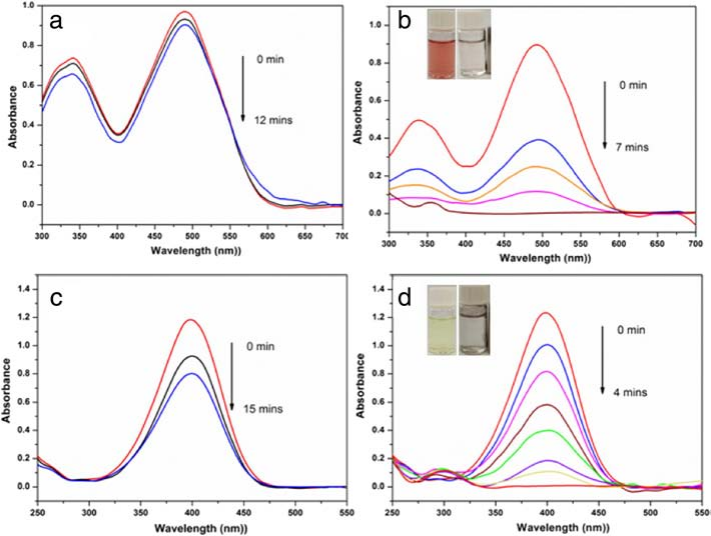
Successive UV–vis absorption spectra of reduction of Congo red (**a**, **b**) and 4-nitrophenol (**c**, **d**) catalyzed by HNTs@Fe_3_O_4_@Au and HNTs@Fe_3_O_4_@Au-Ni nanocatalysts, respectively. The *insets* are the digital images of the degradation phenomenon reduced by the corresponding catalysts

To know whether the as-prepared magnetic nanocatalysts possess high catalytic activities, we first investigate the HNTs@Fe_3_O_4_@Au and HNTs@Fe_3_O_4_@Au-Ni nanocomposites by the degradation of Congo red in the presence of NaBH_4_ in water solutions at room temperature. As shown in Fig. 5a, b, similar to the reported result [28], the addition of NaBH_4_ to the solutions of CR dye shows no appreciable change in the absorbance of solution, indicating that BH_4_ ions cannot reduce the CR dyes [29]. After adding HNTs@Fe_3_O_4_@Au into the solution, the absorbance is slightly decreased, but no obvious decoloration of CR dye is observed within 7 min. This indicates that the HNTs@Fe_3_O_4_@Au nanocomposite has feeble catalytic action to the decoloration of CR dye. However, when the HNTs@Fe_3_O_4_@Au-Ni nanocomposite is added to the CR solution containing BH_4_ ions, the absorbance is rapidly decreased and the color of solution almost becomes colorless within 7 min. As we mentioned above, Janus nanoparticles usually show interesting catalytic activities which is determined by its unique structures. Here, the Janus Au-Ni NPs contained nanocatalyst which reveals excellent degradation activity, which can prove that the specificity of the Janus structure may play an important role in the catalytic reactions again.

To know whether the as-prepared magnetic nanocatalysts possess broad-spectrum catalytic activities, we then investigate the HNTs@Fe_3_O_4_@Au and HNTs@Fe_3_O_4_@Au-Ni nanocomposites by the reduction of 4-nitrophenol in the presence of NaBH_4_ in water solutions at room temperature, which is a well-known model reaction and has been widely used to evaluate the catalytic rate of noble metal catalysts [30]. For comparison, the dosage of both nanocatalysts is the same, the UV–vis spectra at different time (*t*) are shown in Fig. 5c, d. In the absence of any catalysts, the characteristic peak at 400 nm ascribed to 4-nitrophenol remains unaltered even when a large excess of NaBH_4_ is added. After the addition of HNTs@Fe_3_O_4_@Au catalyst, the peak at 400 nm decreases slowly with time and the peak does not show remarkable decline even after 15 min, while the catalytic activity can be enhanced by employing the Janus Au-Ni nanoparticles. After the reduction by the HNTs@Fe_3_O_4_@Au-Ni catalyst, the peak at 400 nm decreases gradually with time, while a new peak appears at 295 nm due to the formation of 4-aminophenol. The color of the reaction system changes from bright-yellow to colorless after the addition of HNTs@Fe_3_O_4_@Au-Ni for 4 min, indicating the complete reduction of 4-nitrophenol.

Anisotropic catalyst particles may also enable self-propellant microengines that can be fueled by bubbles generated by spatially controlled catalytic reactions [31]. These results demonstrate that the Au-Ni contained nanocomposites are superior catalysts than Au itself and other supported Au catalysts, presumably attributed to the electronic junction effect of Au and Ni NPs [27, 32, 33]. This electronic junction effect can also be observed in reduction catalysis of H_2_O_2_ by Au-Fe_3_O_4_ dumbbell-like structure [32]. In addition, the Au NPs in Janus structure are stable against aggregation during harvest procedure, resulting in the enhanced degradation of organic dyes.

Furthermore, as reported, the catalytic activities of flower-like Au-Fe_3_O_4_ is lower than that of Janus structures, which shows that Janus specificity may play important effect through the whole degradation process. Although the Au/Ni alloy NPs were not yet prepared in this report, we can rationally deduce that the catalytic efficiency of Au/Ni alloy NPs is lower than that of Janus-like structures [27], presumably due to that, the Au surfaces in alloy NPs are mainly occupied by the Ni adulterants and thus suppress the reaction rate of dyes.

**Fig. 6:**
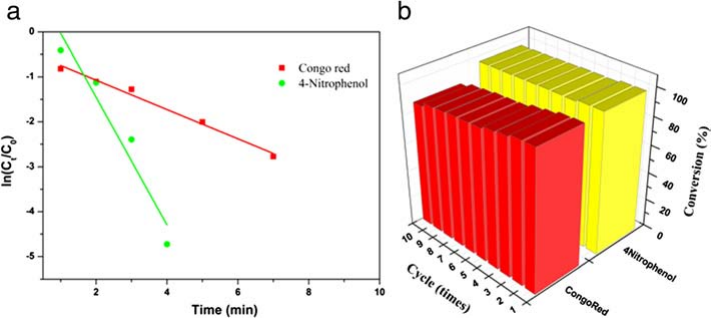
**a** Plots of ln(*C*_*t*_/*C*_*0*_) against time for the reduction of Congo red and 4-nitrophenol with HNTs@Fe_3_O_4_@Au-Ni nanocatalyst. **b** Conversion of the above dyes in 10 successive cycles of reduction and magnetic separation with HNTs@Fe_3_O_4_@Au-Ni nanocatalyst

From the above discussion, we can note that the concentration of NaBH_4_ greatly exceeds that of the dyes and the nanocatalysts and the reduction is mainly catalyzed by the HNTs@Fe_3_O_4_@Au-Ni NCs. Therefore, the kinetics of this reduction can be treated as pseudofirst-order to 4-nitrophenol concentration [34]. The kinetic equation of the reduction can be expressed as

1 $$ \ln \left({C}_t/{C}_0\right)=-{K}_{\mathrm{ap}}t $$

where *C*_0_ is the initial absorbance of the dyes at the maximum absorption wavelength, *C*_*t*_ is the absorbance of dyes at maximum absorption wavelength under different time *t*, and *K*_ap_ is the apparent rate constant. The plot of ln(*C*_*t*_*/C*_*0*_) against *t* should give a straight line with slope *K*_ap_. As seen in Fig. 6a, the plots display a straight line, indicating the good coincidence with the pseudofirst-order equation and suggesting that the nanocatalyst owns high catalytic activity and efficiency. In addition, the as-prepared nanocatalysts show both catalytic and magnetic properties which can be easily recycled by an external magnet after the catalytic reduction, and we find that the nanocatalysts still exhibit excellent catalytic activity even after 10 consecutive cycles of magnetic separation-reduction, as shown in Fig. 6b.

## Conclusions

In the present study, the symmetric Au NPs and Janus Au-Ni NPs decorated clay-based nanocomposites have been successfully fabricated by simple, green, and efficient methods, which generate two different magnetic and low-cost heterogeneous nanocatalysts HNTs@Fe_3_O_4_@Au and HNTs@Fe_3_O_4_@Au-Ni. Thus, we have successfully prepared uniformly distributed Au and Au-Ni NPs over the surface of HNTs, and the catalytic efficacy of these NCs has been studied for the reduction of two organic dyes using NaBH_4_ as a model reaction. Interestingly, we find that the Janus structural specificity of Au-Ni NPs decorated nanocatalyst displays remarkable catalytic activity than the isotropic Au NPs decorated nanocatalyst, which demonstrate that the Janus bimetallic Au-Ni NPs indeed play an important role in these degradation reactions. Furthermore, the nanocatalyst can be reused repetitively for these reduction reactions owing to their convenient recovery from the reaction solution through simple adsorption by magnet. Therefore, the HNTs@Fe_3_O_4_@Au-Ni nanocomposite may have a great potential application in the catalyst fields used as recyclable and low-cost catalytic materials.
